# Cellular behavior of L929 and MG-63 cells cultured on electrospun nanofibers of chitosan with different degrees of phosphorylation

**DOI:** 10.1007/s40204-016-0048-4

**Published:** 2016-04-28

**Authors:** Pallab Datta, Asmita Ray

**Affiliations:** Centre for Healthcare Science and Technology, Indian Institute of Engineering Science and Technology, Shibpur, Howrah, 711103 India

**Keywords:** Phosphorylation, Electrospinning, Bone regeneration, Chitosan, MTT assay

## Abstract

Phosphate groups chemically grafted onto polymer substrates can be used as biomimetic analogs for in vitro studying of function of biomacromolecules and also as tissue substitutes in clinical conditions of organ loss. Despite this inspiration, studies correlating effect of degree of phosphate grafting of a polymer on fabrication and biological properties of polymers are lacking. In this work, *N*-methylene phosphonic chitosan (PC) with different degrees of phosphate contents were synthesized and the effect of phosphate grafting on electrospinning behavior of substituted polymers is investigated. In PC, higher phosphate content widened concentration range for nanofiber formation. Balance between conductivity and viscosity of solutions played a determinant role in the success of electrospinning process. Culture of L929 cells showed grafting-dependent increase in cell proliferation. On the other hand, culture of MG-63 cells showed a positive correlation between grafting degree and Alkaline Phosphatase (ALP) expression. It is concluded that improvement of cell response parameters of nanofiber scaffolds can be attained as a function of controlled degree of phosphate grafting in polymeric biomaterials with implications for bone tissue engineering applications. Such studies may also be useful to develop quantitative structure activity relationships of functional polymers.

## Introduction

Phosphorous containing polymers have shown potential to be used as functional polymers for drug delivery, regenerative medicine and tissue engineering applications. Phosphates possess unique ionization properties which render biological macromolecules resistant to non-enzymatic hydrolysis, but susceptible only to specific enzymatic action (Chang and Lim 1998; Monge et al. 2011; Westheimer 1987). Interestingly, phosphorous-based compounds exert their functions both in the soluble and insoluble in biological systems. Thus phosphorous modifications of synthetic polymers can become a feasible approach for design and synthesis of biomimetic polymers for various applications.

For example, phospholipid mimetic 2-methacryloyloxyethyl phosphorylcholine (MPC) has been synthesized as biomembrane structural analog and has shown success in reducing protein denaturation on surfaces and improving bio-compatibility of artificial organs (Ishihara 2000; Junji and Kazuhiko 2003). This modified polymer is also shown to exhibit anti-fouling properties and used in soft contact lens (Goda and Ishihara 2006). Poly(vinyl alcohol) phosphate esters have been synthesized as polynucleotide analogs or as phospholipid mimetic taste sensing materials (Majumdar and Adhikari 2006; Yu and Carlsen 2008). Similarly, polymer derived from phospholipid monomer 1-palmitoyl-2-[12-(acryloyloxy)dodecanoyl]-*sn*-glycero-3-phosphorylcholine exhibits reduced platelet deposition and has been applied as biomaterial for cardiovascular repair (Marra et al. 1997). Phosphate-based polymers have also received critical attention for their role in biomineralization process for bone tissue engineering. This is inspired from the natural biomineralization process wherein phosphate moieties attached to phospholipids, phosphoproteins and phopsphoproteoglycans act as nucleation sites for hydroxyapatite formation and also control mineral morphologies (Collier and Messersmith 2001; George and Veis 2008; He et al. 2005; Hunter and Goldberg 1993; Wise et al. 2007). In addition, many regulatory signals of the body are controlled by phoshphorylation of key proteins either present in form of growth factors or matrix-bound scaffold proteins (Alessi et al. 1997; Good et al. 2011; Van Hoof et al. 2009). Apart from above applications, phosphate groups grafted to polymer backbones have also been demonstrated to improve function of osteoblast cells and augment performance of bone grafts (Sailaja et al. 2009; Xu et al. 2011). In our previous studies, we have also observed that phosphate groups grafted on polyvinyl alcohol and chitosan show markedly improved osteoblast like cell proliferation compared to their parent non phosphorylated polymers (Datta et al. 2013). In this study, we further wanted to investigate if the improvements in cell functionalities are correlated with the degree of phosphate modification and what types of relationship exist between the polymers modified to different degrees of phosphate content and cell behavior.

From literature, it can be found that there are only limited number of studies which report relationship between extent of phosphate grafting with physico-chemical or biological properties relevant for tissue engineering. Such correlation studies with degree of substitutions would be necessary for effective translation into clinical products. Parallely, it is also important to note that along with material chemistry, structural and textural properties of fabricated biomaterials also determine cell response. In this respect, amongst the different methods available for material fabrication, electrospun nanofibrous scaffolds closely resemble the attributes of natural extracellular matrix and are ideal for tissue engineering applications (Liao et al. 2006). However, electrospinning of a polymer is governed by interplay of intrinsic polymer properties like polymer crystallinity, solution viscosity, conductivity, surface tension and dielectric constant, making optimization of the process a critical task (Bharadwaj and Kundu 2010; Tang et al. 2010). Correlating changes in physical properties with extent of chemical modification is, therefore, crucial for development of nanofiber scaffolds based on phosphorylated polymers.

In this study, derivatives of chitosan with different degrees of phosphorylation were synthesized, characterized and electrospinning behavior of various solutions containing these derivatives was compared. Finally, nanofibers produced were characterized for their biocompatibility by culture with L929 and MG 63 cells.

## Experimental

### *N*-methylene phosphonic chitosan

Phosphorylation of chitosan was undertaken by means of coupling reaction of amino group with phosphonates in the presence of formaldehyde as reported earlier with certain modifications [32, 33, 42]. Briefly, 3 % w/v chitosan (grade <90 % deacetylated, M/s Marine Chemicals, Cochin) solution was reacted with phosphorous acid and formaldehyde (one part by weight). Phosporous acid and formaldehyde were obtained from M/s Himedia Laboratories, India. The total volume of solution was 30 ml. Reaction was conducted at 70 °C. To obtain different degrees of phosphorylation, reaction was carried out for 3.5, 7 and 14 h. Gel-type precipitate was obtained by adding excess anhydrous ethanol and vacuum dried at 50 °C. Unreacted substances were washed off 4–5 times by repeated washing procedure in which dried product at each stage was dissolved in water, precipitated with excess ethanol, centrifuged at 7000 rpm and supernatant discarded.

### Solid-state characterization

FTIR spectrum of original polymers and their phosphorylated derivatives were acquired in a GX spectrophotometer (Jasco, Japan).

### Solution property measurements

pH and conductivity were measured by Thermo Okaton. Surface tension was measured by laboratory stalagmometer.

### Dilute solution viscometry

Solutions of various concentrations in the range of 0.1 and 0.5 g dl^−1^ were filled in an Ubbelohde capillary viscometer and equilibrated for 15 min in a water bath at 25 °C. Run time was measured thrice for calculation of reduced viscosity and intrinsic viscosity. Intrinsic viscosity was then used to calculate the molecular weight of polymers based on Mark Howink constants with the values of *K* and *a* being 1.81 × 10^−3^ and 0.93, respectively (Ramos et al. 2003).

### Electrospinning

Polymer solutions of varied compositions were subjected to electrospinning on a conventional set up. Syringe assembly was fit into syringe pump (Physics Equipment, Chennai, India) and a collector consisting of copper plate covered with aluminum foil was placed 10 cm downward from tip of the needle. A voltage of 20 kV between the two electrodes was maintained. These procedural parameters were adapted from previous report (Datta et al. 2012). Flow rate was varied between 0.25 and 10 µl min^−1^. Various concentrations (starting from 9:1 parts to point beads free nanofibers were obtained with total polymer concentration of 8 % w/v) of polyvinyl alcohol: chitosan or substituted chitosan were studied. For cell culture studies, a 7:3 mixture of PVA: chitosan was used. Polyvinyl alcohol was obtained from Sigma Aldrich (99 % + hydrolyzed; average *M*_w_ 130,000 grade) and used as received.

### Scanning electron microscopy

The samples were observed under EVO 60 scanning electron microscope (Carl Zeiss SMT, Germany) after gold coating. Glutaraldehyde (50 mM) solution (Himedia, India) was used for cross linking the fibers (4 h, at Room temperature 25 °C) for biological studies as reported (Datta et al. 2012) and washed thoroughly in acetone and ethanol to remove unreacted glutaraldehyde. Resultant nanofibers were subjected to image analysis for nanofiber diameter measurements (Oznergiz et al. 2014).

### Biocompatibility of phosphorylated derivatives

L929 fibroblast and Pre-osteoblast-like MG63 cells (NCCS, Pune, India) were cultured in DMEM complete media with 10 % FBS (Himedia, India) as previously reported [24] in 37 °C, humidified environment (Esco, Singapore). Cell counts were standardized.

Samples (3 each) were sterilized in 70 % ethanol followed by UV sterilization with 30 min treatment, placed in 24-well tissue culture polystyrene plates and soaked in culture medium overnight. Cells were seeded at density of 10^5^ cells/cm^2^ in each well plate. Viability was assessed using 3-(4,5-dimethylthiazol-2-yl)-2,5-diphenyltetrazolium bromide (MTT) assay taken on days 3, 5 and 7 as per procedures described in previous works. After predetermined time intervals, media was discarded from cel-seeded scaffolds followed by washing with PBS thrice and incubation with 200 μl of 5 mg ml^−1^ MTT solution (Sigma) at 37 °C for 4 h. The formazan crystals so formed were dissolved in Dimethyl sulfoxide (DMSO) and optical activity measured at 570 nm. For each type of scaffold, a reading without any cell incubation was taken as blank and used to subtract from cell seeded scaffold readings. Absorbance was read in 96-well plates on a microplate reader at 570 nm. For determination ALP activity, on day 3 MG-63 cell homogenates were prepared and incubated with p-nitrophenyl phosphate at 37 °C. p-nitrophenyl released by the enzyme was then measured spectrophotometrically and calculated against a standard curve of pNP.

### Immuno-cytochemical (ICC) analysis of Ki67 expressions

L929 cells were fixed with 4 % paraformaldehyde for 10 min at 25 °C for ICC assay. Samples were incubated with 10 % goat serum for 30 min to block non-specific binding of the antibodies. Cells were then incubated with primary antibodies. Ki67 expression was observed on L929 cells. A dilution of 1:500 was used for ki67. Alexa Fluor 596 conjugated secondary antibody was used. Cells were counterstained with 4′,6-diamidino-2-phenylindole (DAPI). All reactions were performed in dark at room temperature (25 °C).

### Image acquisition

The digital images were grabbed by Nikon inverted fluorescence microscope (Nikon eclipse Tί, Japan) at 20× magnification and green filter for Fluorescein isothiocyanate (FITC) and blue filter for DAPI under 20x objectives (NA 0.8). Field of view for image was 690 × 515 μm^2^ and pixel resolution was 0.17 µm.

## Results and Discussion

### Synthesis of different degrees of phosphorylated chitosan

Phosporylated polymers have shown benefits in many cell-based assays for tissue engineering applications. However, there is not much information available on correlation of physicochemical or biological properties with degree of phosphorylation in the polymer. Such quantitative correlations are important for understanding structure–activity relationship of a polymer for tissue engineering as well as to develop standards for clinical applications. Phosphorylated chitosan is a polymer with previously reported potential for bone tissue differentiation (Lopez-Perez et al. 2010). Phosphorylated chitosan in form of *N*-methylene phosphonic chitosan (PC) with different degree of phosphorylations was obtained via the Kabachnik-Fields Reaction—a widely reported strategy used in synthesis of peptidomimetic compounds in area of synthetic biology, as well as phosphopeptide research (Naydenova et al. 2009). Chitosan has close resemblance with glycans of tissue extracellular matrix, making it possible to obtain functional mimetic compounds of the natural macromolecules by this reaction (Lebouc et al. 2005) since it is also reported that phosphate groups play important role in functionalization of many glycans (Takashiba et al. 2006). In the mechanism, phosphorous acid reacts with amino moities to form a complex which further reacts with formaldehyde to form an adduct. In the second step adduct is converted to aminomethyl phosphonates (Cherkasov and Galkin 1998). Reaction was continued for 3.5, 7 and 14 h yielded a product with elemental composition of C (28.31 %), N (7.78 %), O (57.38 %), P (6.61 %); C (28.22 %), N (7.48 %), O (56.64 %) and P (7.67 %); and C (27.1 %), N (6.79 %), O (57.87 %), P (8.24 %) and were designated as PC-1, PC-2 and PC-3, respectively, in this study. An increase in degree of substitution was confirmed by examining P/N ratio in PC-1 (1.02), PC-2 (1.21) and PC-3 (0.85).

### Characterization of products

**Fig. 1:**
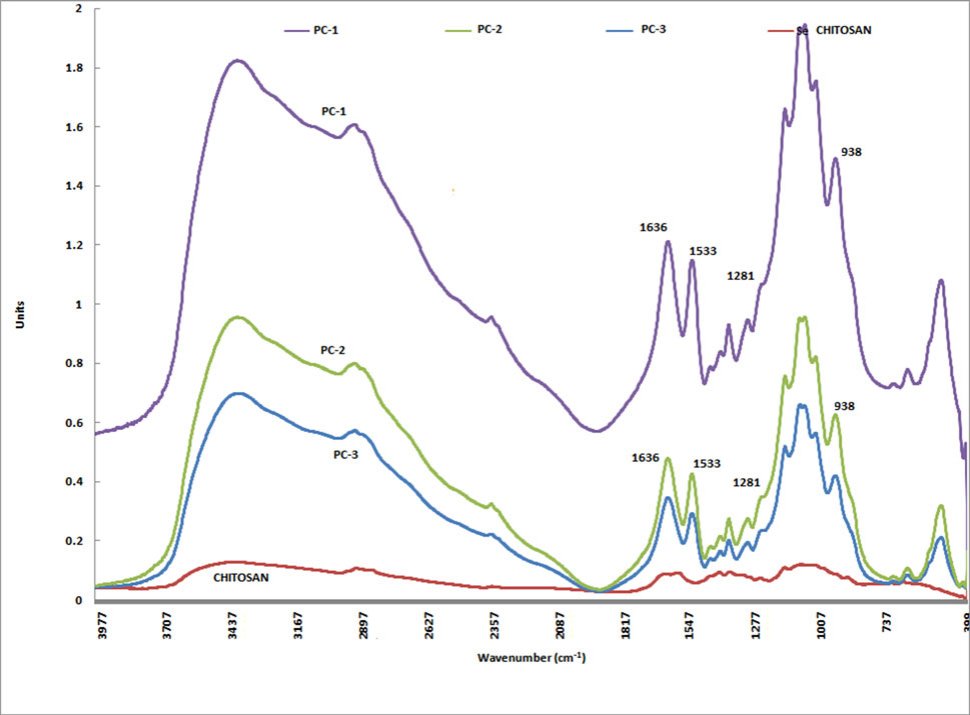
FTIR spectra of Chitosan, *N*-methylene phosphonic chitosan PC-1, PC-2 and PC-3

PC vibrations bands are shown in Fig. 1 wherein 1636 and 1533 cm^−1^ bands are attributed to antisymmetric and symmetric vibrations of secondary amines in aminomethyl phosphonic derivative while absorptions related to phosphorous containing bonds were observed at 1281 and 938 cm^−1^. For characterization, area under curve (AUC) under the peak at 938 was calculated and found to be PC-1, PC-2 and PC-3, 0.166, 0.389, 0.628 and 1.665, respectively, for chitosan, PC-1, PC-2 and PC-3 samples showing an increase with extent of substitution reaction.

### Solution state properties

The 1 % w/v PC-1, PC-2 and PC -3 aqueous solutions showed conductivities values of 1034, 702 and 659 µS/cm, respectively, while surface tension of these solutions were recorded to be 61.4, 60.1 and 58.4 mN/m, respectively. The molecular weights of PC-1, PC-2 and PC-3 were determined to be 207, 161 and 129 kDa, respectively (Table 1). In PC, anionic phosphate groups replaced positively charged amino groups with which may neutralize each other to certain extent resulting in the observed decrease solution conductivity. Decrease in molecular can be attributed to chain scission during reaction. However, most present phosphorylation strategies for chitosan modification are accompanied by some decrease in molecular weight of chitosan and hence the effect of molecular weight on solution properties could not be completely eliminated.

**Table 1:** Effect of degree of phosphorylation on physicochemical properties of polymer solutions

	Conductivity (µS/cm)	Surface tension	Viscosity (mPa s)	*M*_v_
Chitosan	1540 ± 16.1	62.5 ± 0.7	20.6 ± 1.66	245
PC-1	1034 ± 22.3	61.4 ± 2.2	15.6 ± 0.98	207
PC-2	702 ± 12.5	60.1 ± 2.9	7.63 ± 2.45	161
PC-3	659 ± 7.9	58.4 ± 3.1	4.17 ± 1.99	129

These results are in concurrence with a previous report wherein internal neutralization in phosphonic chitosan has been shown to result in decrease in conductivity and similar effect on surface tension has also been reported due to structural changes in chitosan solutions (Rodriguez et al. 2005).

### Electrospinning behaviors

**Fig. 2:**
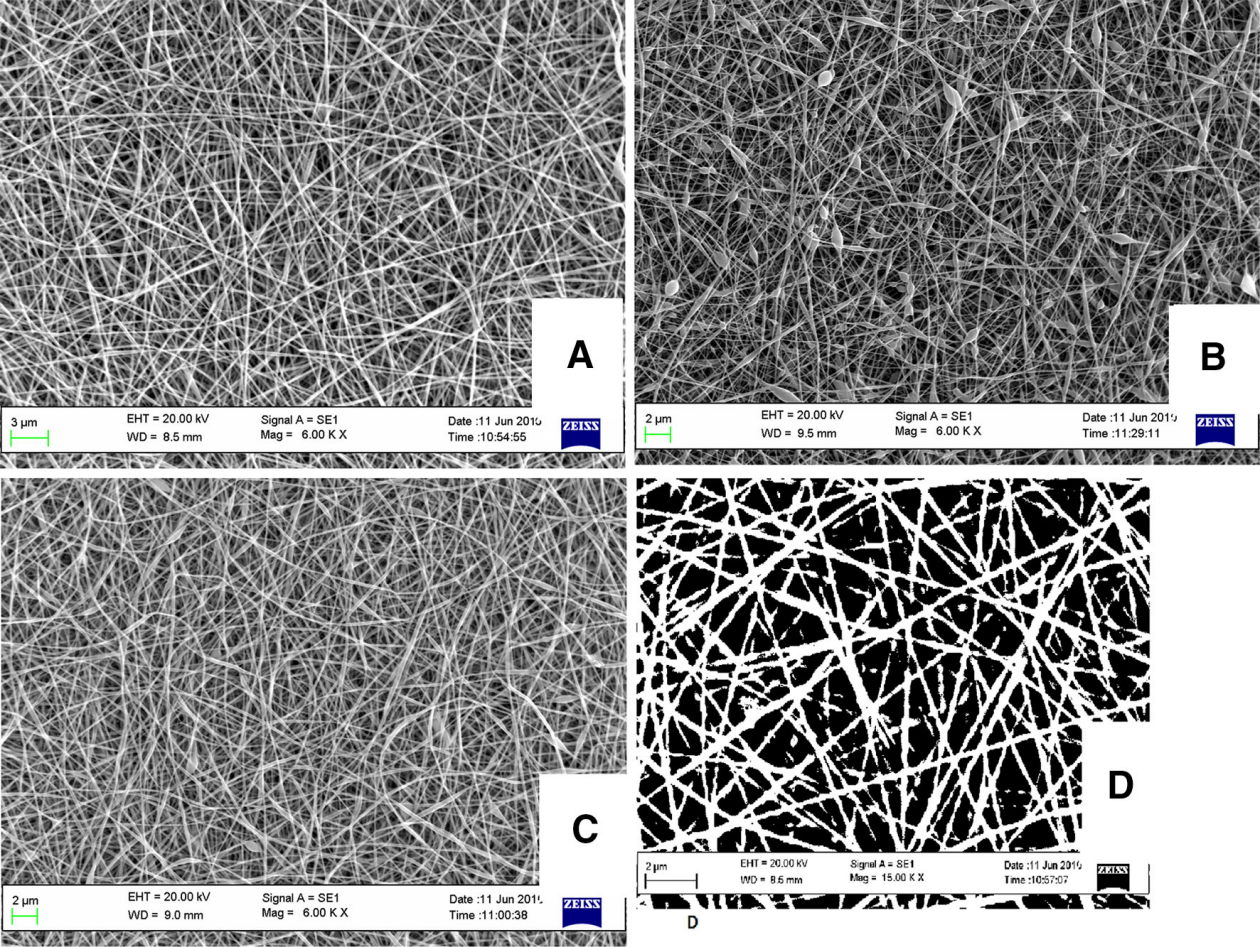
SEM micrographs of as spun deposits of **a** 8 % w/v polymer with 5:5 ratio of PVA:PC-2, **b** with 4:6 ratio of PVA:PC-2, **c** with 4:6 ratio of PVA:PC-3 solution where defect-free fibers are obtained and **d** SEM micrograph after applying image analysis for **a**

In a previous paper, it has been reported that standalone nanofibers of PC by electrospinning cannot be fabricated and hence necessity for addition of PVA as an electrospinning aid was emphasized (Datta et al. 2012). Some parameters of electrospinning were optimized for a single substituted chitosan polymer. In this work, we varied only the polymer composition and kept all other parameters of electrospinning constant so as to get the difference between the spinning behavior arising due to different degrees of phosphate groups in the polymers. Amongst PC-1, PC-2 and PC-3 we found 3:7 ratio of PC-1:PVA to produce beaded morphology while in case of PC-2 and PC-3 the highest ratio in which defect-free fibers could be generated was 5:5 and 6:4, respectively. This indicated that phosphorylation tended to favor the electrospinning process as bead-free morphology is now obtainable with less PVA content (Fig. 2a, b and c).

The changes in electrospinning behavior could be explained by examining properties of PC-1, 2 and 3 at the concentration boundaries at which electrospinning was feasible in each substituted polymer as is represented in Table 2. As seen from table, PC-1:PVA mixed in the ratio of 3:7 (800 µS/cm; 0.78 Pa s) and PC-2:PVA at ratio of 5:5 (838 µS/cm and 0.91 Pa s) were electrospinnable whereas PC-2/PVA at 6:4 (1012 µS/cm, 0.88 Pa s) ratio produced beads on string fiber morphology. However, a PC-3/PVA at the same ratio was electrospinnable. Thus it can be concluded here that conductivity and viscosity played more determinant roles for morphology of fibers produced.

**Table 2:** Physicochemical properties of phosphorylated polymer solutions at concentration boundaries of electrospinning

	Conductivity (µS/cm)	Surface tension (mN/m)	Viscosity (Pa s)	Electrospinnability
PC-1/PVA (3:7)	1275	49.61	2.65	Yes
PC-2/PVA (5:5)	843	48.65	1.88	Yes
PC-3/PVA (6:4)	734	46.13	0.78	Yes

Second, for solutions with high conductivities due to phosphorylation, a higher solution viscosity was also required to attain balance between the forces responsible for successful electrospinning. A surface with high charge density increases tendency of polymer chain to repel each other and when not counter-balanced by the increase of viscosity, prevents sufficient chain entanglements necessary to produce bead-free fibers. In case of PC, degree of phosphorylation decreased solution conductivity without affecting entanglement viscosity in polymer and hence increasing electrospinning window for PC. It can thus be conclusively stated that phosphorylation primarily affects the electrospinning behavior by altering solution conductivities of polymers. The higher solution conductivities, however, can be offset if phosphorylation is also accompanied by an appropriate increase in viscosity of solutions. This understanding can form the basis of selecting further electrospinning parameters to obtain defect-free nanofibers of phosphorylated polymers. The diameter of the nanofibers was found to be 185 ± 25, 225 ± 40 and 198 ± 30 for PC-1, PC-2 and PC-3 showing no significant differences between them. Figure. 2d depicts the representative SEM images which were obtained for PC-1 nanofibers to calculate diameters.

### Biocompatibility

**Fig. 3:**
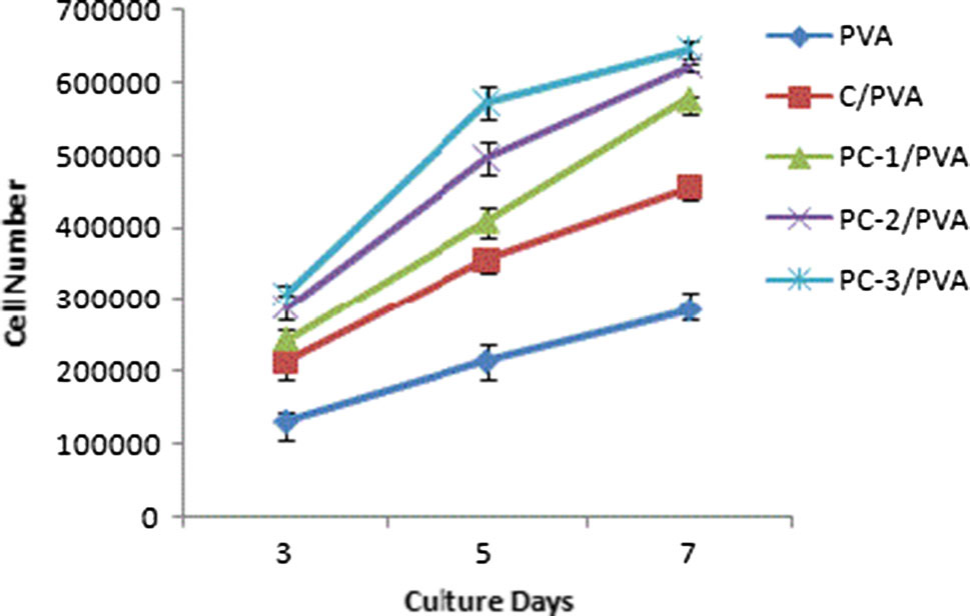
MTT dye reduction assay on day 3 through 7 of L929 cells cultured on nanofiber sheets as a function of different degrees of phosphorylation (DP)

**Fig. 4:**
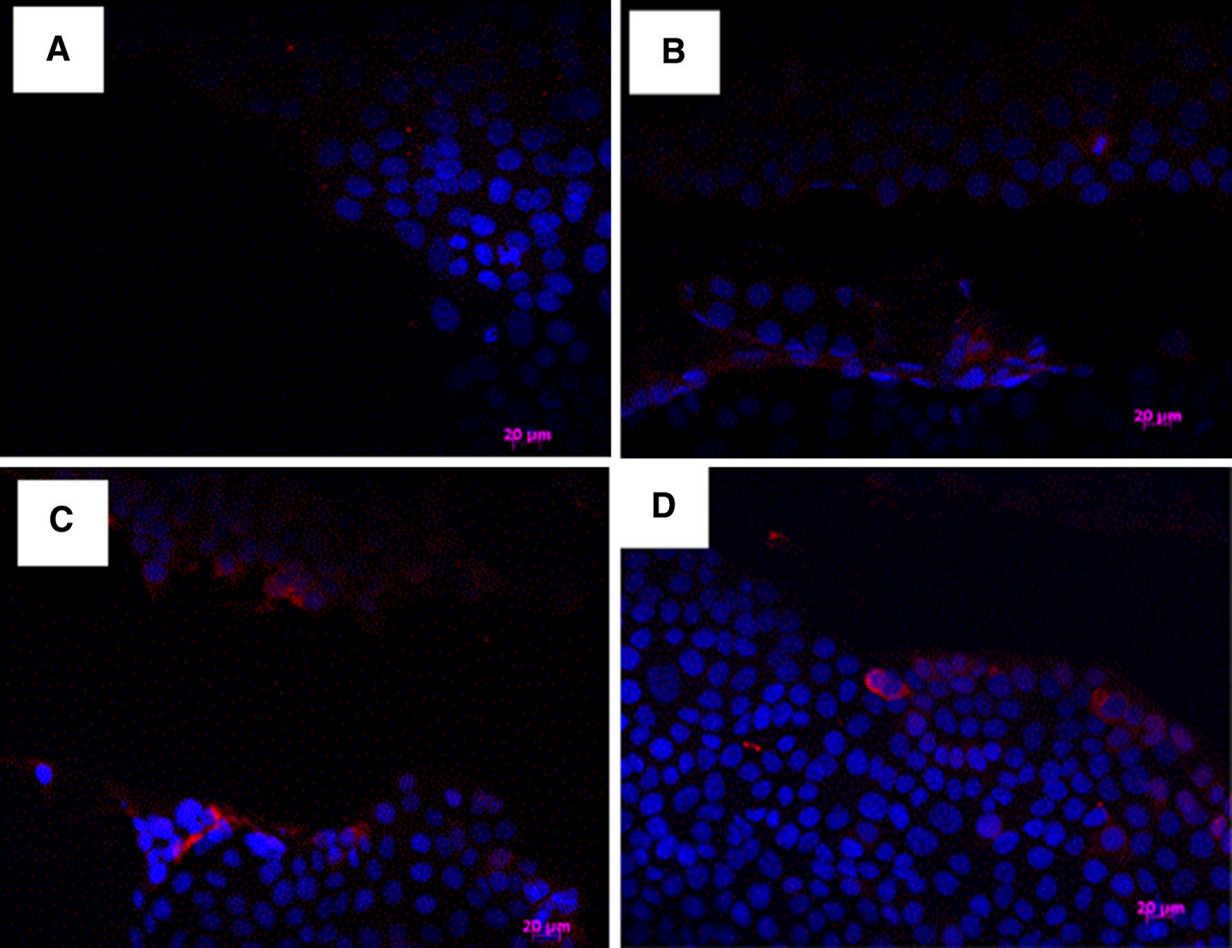
Fluorescence microscopic images (×20) showing expression of Ki67 in L929 cells on different study groups **a** PC-3, **b** PC-2, **c** PC-1 and **d** Chitosan. The expression was comparatively higher in PC3 group in comparison to control (C) and other two study groups. Increasing expression of Ki67 marker from PC3 to PC2 to PC1 study groups is indicating that increased degree of phosphorylation has positive impact on cellular proliferation which is evident from increased expression of proliferation marker

The effect of degree of phosphorylation in chitosan on cell viability was first evaluated by culturing L929 cells as they are easy to maintain and had been known to produce high correlation with results of animal bioassays for which they are recommended in ISO biocompatibility procedures. As seen from Fig. 3, % cell viability relative to PVA nanofibers on chitosan/PVA, PC-1/PVA and PC-2/PVA and PC-3/PVA nanofibers was found to be 166, 191, 231 and 268 % res. Thus in comparison, cell proliferation was found to be more in polymer with higher degree of phosphorylation as there existed a significant difference between them (*p* < 0.05) with almost a linear correlation between phosphate substitution and cell proliferation. To examine the proliferation of L929 cell on phosphorylated electrospun scaffolds, Ki67 expression was observed by immuno-cytochemical analysis. Data revealed that Ki67 expression is significantly increased with degree of phosphorylation as shown in Fig. 4.

**Fig. 5:**
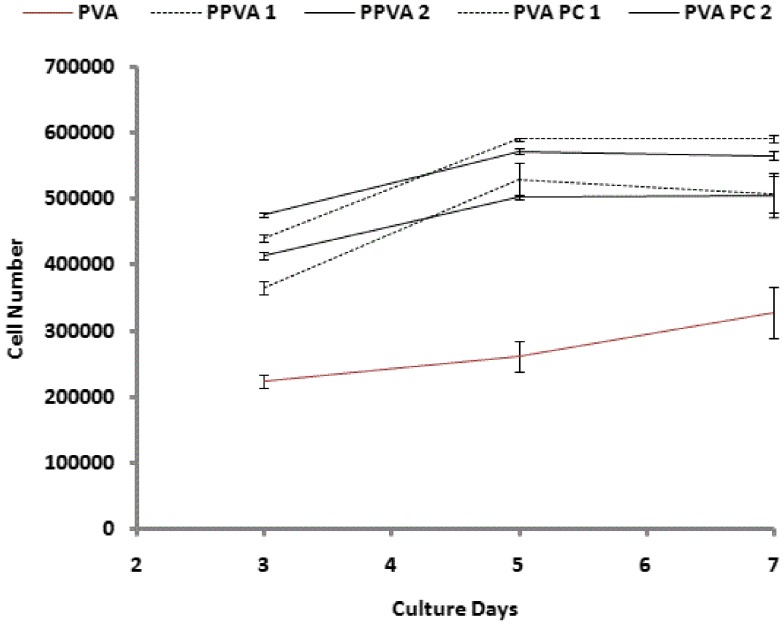
MTT dye reduction assay on day 3 through 7 of MG-63 cells cultured on nanofiber sheets as a function of different degrees of phosphorylation (DP)

For application of bone tissue engineering, MG-63 cell line was chosen as the in vitro cell model because they express characteristics similar to osteoblasts and hence are recommended for evaluating bone tissue engineering scaffolds [24]. However, in between chitosan and its phosphorylated derivatives, the role of phosphates in influencing MG-63 viability was not observed even after 7th day of culture. The viability of nanofibers with higher degree of substitution was not significantly different from nanofibers of lower degree of phosphorylation (*p* > 0.05) (Fig. 5).

**Fig. 6:**
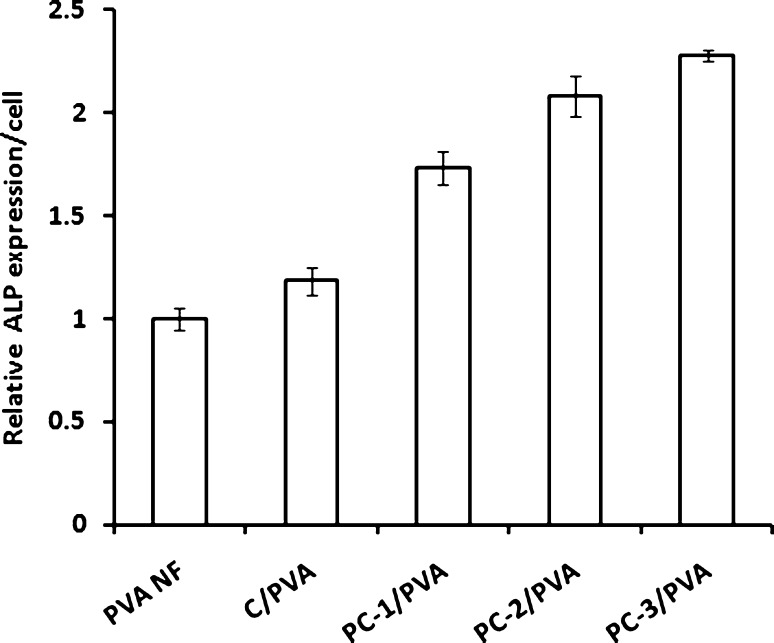
Alkaline phosphatase (ALP) enzyme activity on day 3 of MG-63 cells cultured on nanofiber sheets as a function of different degrees of phosphorylation

In addition to cellular proliferation, effect of degree of phosphorylation was also studied on expression of ALP activities of MG-63 cells (Fig. 6). Results obtained showed that ALP activity for PC-1, PC-2, PC-3 were found to be 1.73 ± 0.08, 2.08 ± 0.09 and 2.28 ± 0.02 units relative to ALP activity of cells cultured on chitosan and normalized to the number of cells on each nano665-fibers illustrating the role of phosphate groups in guiding functional status of cultured cells. It can be noted here that number of viable cells on PC-2 and PC-3 were found to be similar to each other, thus emphasizing the positive role of phosphorylated matrix on enhancing cellular function. These results emphasize that though matrix phosphate may have beneficial effect on cell functions, effect of degree of phosphorylation may be observed based on the cell type.

In the work of Gemeinhart et al., surface modification with increasing phosphate contents also showed 30 % degree of substitution as optimum for cell growth and proliferation (Gemeinhart et al. 2006). Polymers with new properties by phosphate grafting were also demonstrated by Ko and Ma with poly(ethylene-*co*-acrylic acid) surfaces which showed lower water contact angles than the initial polymer attributed to highly mobile and hydrophilic phosphate-chains by the authors (Ko and Ma 2009).

## Conclusions

This work demonstrates a technique to successfully graft phosphate groups onto organic polymer surfaces in a biocompatible aqueous environment, which may open new avenues to functionalize synthetic polymeric and natural macromolecule-derived biomaterials. Importantly, the study demonstrates that degree of phosphorylation substitution can be correlated with cellular functions and the level of correlation may be different for different types of cells. In this light, this work emphasizes the need for studying quantitative structure activity relationships in modified polymers for tissue engineering applications.
